# Quantitative proteomics reveals role of sugar in decreasing photosynthetic activity due to Fe deficiency

**DOI:** 10.3389/fpls.2015.00592

**Published:** 2015-08-03

**Authors:** Sajad M. Zargar, Ganesh K. Agrawal, Randeep Rakwal, Yoichiro Fukao

**Affiliations:** ^1^Centre for Plant Biotechnology, Division of Biotechnology, S K University of Agricultural Sciences and Technology of KashmirSrinagar, India; ^2^Research Laboratory for Biotechnology and BiochemistryKathmandu, Nepal; ^3^GRADE Academy Private LimitedBirgunj, Nepal; ^4^Faculty of Health and Sport Sciences and Tsukuba International Academy for Sport Studies, University of TsukubaTsukuba, Japan; ^5^Department of Bioinformatics, Ritsumeikan UniversityShiga, Japan

**Keywords:** Arabidopsis, proteomics, Fe deficiency, sugar, photosynthesis

## Importance of iron in plant

Iron (Fe) is an essential micronutrient and its deficiency is a serious nutritional problem for all living organisms. This is because Fe is not only a basic requirement in cellular functions such as the redox reactions in photosynthesis and respiration, but is also required in the enzymatic processes like DNA replication, lipid metabolism, and nitrogen fixation in plants (Lan et al., 2011; Briat et al., 2015). As the photosynthetic apparatus contains much Fe, involved in many metabolic reactions in plastids, it becomes an important factor for survival of green plants. In plants, Fe deficiency can be observed by the development of chlorosis, which reduces the photosynthetic activity (Spiller and Terry, 1980; Terry, 1980; Straus, 1994; Briat et al., 2015).

## Proteomics studies related to iron deficiency

Proteomics is being increasingly used to expand our understanding of plant growth and development under both normal and stressful environmental conditions (Agrawal and Rakwal, 2008). Proteomic technology has also been employed as a powerful tool in the elucidation of metabolic rearrangements caused by Fe deficiency (López-Millán et al., 2013). Recently, quantitative proteomics approach was applied to understand the impact of Fe deficiency on plant metabolism; combined with physiological studies, the impact of Fe deficiency on photosynthesis was discerned (Zargar et al., 2013, 2015). Fe deficiency is known to alter both chloroplast structure and photosynthetic rate in higher plants as it alters the chlorophyll synthesis (Briat et al., 2015). The comparative proteome analysis of chloroplast thylakoids explains the plasticity of thylakoid membranes in response to Fe deficiency (Andaluz et al., 2006). A phosphoproteomic study of the thylakoid membrane proteome, from Fe-sufficient and Fe-deficient plants identified several proteins with post-translational modifications, that included, the doubly phosphorylated form of the photosystem II oxygen evolving complex, PSBH, ascorbate peroxidase, peroxiredoxin Q, and two major LHC IIb proteins (LHCB1 and LHCB2) (Laganowsky et al., 2009). Lan and coworkers have used the iTRAQ method to examine protein regulations involved in Fe homeostasis in Arabidopsis shoots (Lan et al., 2011). The abundance of 45 phosphoproteins was significantly changed upon Fe deficiency, which includes kinase A/calcium calmodulin-dependent kinase II, casein kinase II, and proline-directed kinase, indicating a possible critical function of these kinase classes in Fe homeostasis (Lan et al., 2012).

Recently, we applied the iTRAQ-OFFGEL method for understanding impact of Fe deficiency on photosynthesis and to unravel the proteome underlying the cross-talk between Fe deficiency and excess Zn in Arabidopsis (Zargar et al., 2015). Results revealed that Fe deficiency might lead to disruption of sugar synthesis and utilization.

## Iron deficiency influences the photosynthetic machinery and sugar levels: Proteomic insights

The impact of Fe deficiency on photosynthesis in Arabidopsis has been very well documented (Zargar et al., 2013). Here we will majorly focus on the role of sugar in decreasing photosynthetic activity due to Fe deficiency. Two sugar transporters, major facilitator super family protein (STP13; AT5G26340) and sugar transporter 4 (STP4; AT3G19930) that have shown higher expression levels under Fe-deficient conditions were identified. STP13 and STP4 protein expressions were increased to 8.179- and 1.968-fold in Fe-deficient condition (Zargar et al., 2015). STP13 is known to be involved in transport of sucrose, glucose, and hexose (Saier et al., 1999; Lemoine, 2000; Norholm et al., 2006), while STP4 is a monosaccharide transporter (Fotopoulos et al., 2003). Further, we observed that the concentration of sucrose, fructose, and glucose were significantly increased in 2-weeks-old shoots of Arabidopsis grown on Fe deficient conditions compared to the control condition (Zargar et al., 2015). Thus, under Fe deficiency, a higher expression of sugar transporters as well as higher sugar concentration in shoots was observed. As such, Fe deficiency leads to accumulation of sugars in shoots, as synthesis and utilization of these sugars were not properly managed.

Past evidences have shown that root glycolytic (Zocchi, 2006; Jelali et al., 2010) and fermentation (Thimm et al., 2001) processes are enhanced under Fe deficiency, leading to sugar accumulation that derives from starch degradation and/or reorientation of photo-assimilate partitioning probably via sorbitol or sucrose (Loescher et al., 1990). Since these two sugar transporters are mainly expressed in roots and vascular bundle in shoots, these transporters may contribute to the transport of sugars from mesophyll cell to vascular bundle for photosynthesis. Fe deficiency decreases photosynthetic activity, and as such sugar synthesis decreases. Therefore, the plant might need higher sugar levels to maintain fundamental metabolisms; hence sugars might be translocated from roots to shoots. Since STP13 was induced under stress condition, and involved in reabsorption of sugars from roots (Yamada et al., 2011), we presume that higher expression of sugar transporters might have a role in increasing sugar levels in shoots to maintain fundamental processes.

## Sensing the role of sugar

The down-regulated proteins due to Fe deficiency mostly include proteins involved in photosynthesis or ribosomal proteins. It has been well known that Fe deficiency largely affects protein synthesis in chloroplasts as compared to the cytoplasm, because chloroplastic mRNA and rRNA levels are significantly reduced (Spiller et al., 1987). In addition, the expression of various genes involved in different metabolic processes including photosynthesis is regulated by the sugar-driven signals (Sheen, 1990; Oswald et al., 2001). The negative correlation between sugar concentration and photosynthetic activity, and photosynthetic genes expression has also been reported earlier (Foyer, 1988; Sheen, 1990; Oswald et al., 2001). Therefore, the lower expression of photosynthetic genes under Fe-deficient conditions may be partly affected by high sugar concentration.

**Figure 1:**
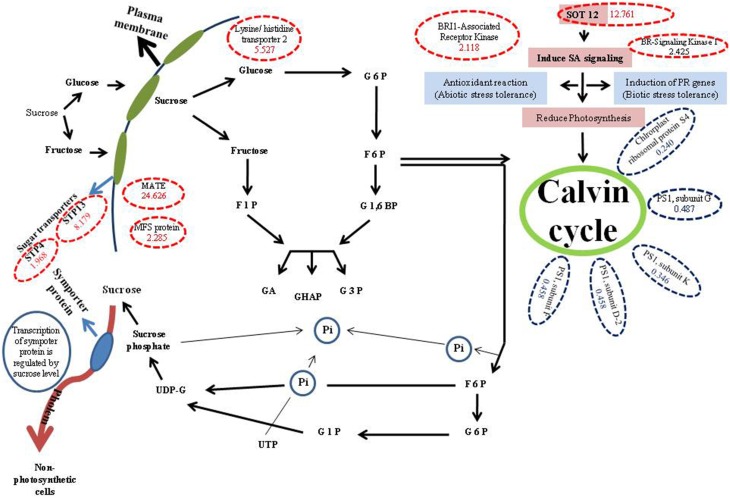
**Metabolic pathway showing role of different proteins involved in synthesis and utilization of sucrose via some proteins with high and low expressions**. G6P, Glucose 6 phosphate; 6PG, 6 phosphogluconate; F6P, Fructose 6 phosphate; F1,6BP, Fructose 1,6 biphosphate; F1P, Fructose 1 phosphate; GA, Glyceraldehyde; DHAP, Dihydroxy acetone phosphate; G3P, Glyceraldehyde 3 phosphate; G1P, Glucose 1 phosphate; UDP-G, UDP-Glucose; SA, Salicylic acid; Red dotted circles show, up regulated proteins (in brackets fold values of proteins in 0-Fe conditions compared to control are demarked); Blue dotted circles show down regulated proteins (in brackets fold values of proteins in 0-Fe conditions compared to control are demarked).

Aforementioned and other key proteins identified in our study were mapped onto metabolic and biological pathways as depicted in (Figure 1), and that explains the possible role of sugars in decreasing photosynthetic activity in Arabidopsis. Based on our results we believe that sugar might have a role in decreasing photosynthetic activity under Fe deficiency conditions. Further, we presume that Fe deficiency in Arabidopsis might lead to reduction in phloem unloading in sink tissues due to which sugars get accumulated in the shoots. Moreover source tissues load solutes into the phloem, but the restricted unloading under Fe deficiency may lead to sugar accumulation, which in turn has a negative effect on the expression levels of proteins involved in photosynthesis. There is also a possibility of sugar signaling involvement in the inhibition of photosynthesis. For example, cells under Fe-deficient conditions lead to decrease in photosynthesis by inducing sugar signaling, which might have role in decreasing expression of proteins involved in photosynthesis. Despite the above evidences and discussion therein, we are of the opinion that further intensive studies will be required linking physiology, biochemical processes with sugar signaling and regulation of genes involved in carbohydrate metabolism, transport, and partitioning.

## Funding

This work was supported by a Grant-in-Aid for Organelle Differentiation as the Strategy for Environmental Adaptation in Plants for Scientific Research of Priority Areas (No. 19039022 to YF) from the Ministry of Education, Culture, Sports, Science and Technology of Japan; a Grant-in-Aid for Scientific Research from Nara Institute of Science and Technology supported by The Ministry of Education, Culture, Sports, Science and Technology, Japan. SZ acknowledges the DBT, New Delhi, India for award of CREST, Overseas fellowship to undertake this research.

### Conflict of interest statement

The authors declare that the research was conducted in the absence of any commercial or financial relationships that could be construed as a potential conflict of interest.
